# Fatigue in adults with congenital heart disease aged over 40 years

**DOI:** 10.1016/j.ijcchd.2025.100601

**Published:** 2025-06-16

**Authors:** Linda Ternrud, Bengt Johansson, David Sparv, Zacharias Mandalenakis, Christina Christersson, Liesbet Van Bulck, Philip Moons, Camilla Sandberg, Joanna Hlebowicz

**Affiliations:** a Department of Cardiology, Skåne University Hospital, Lund, Sweden; b Department of Clinical Science, Lund University, Sweden; c Department of Public Health and Clinical Medicine, Umeå University, Sweden; d Department of Diagnostics and Intervention, Umeå University, Sweden; e Adult Congenital Heart Disease Unit, Sahlgrenska University Hospital, Gothenburg, Sweden; f Department of Medical Sciences, Cardiology, Uppsala University, Sweden; g Department of Public Health and Primary Care, KU Leuven, University of Leuven, Leuven, Belgium; h University of Gothenburg Centre for Person-Centred Care (GPCC), Sahlgrenska Academy, University of Gothenburg, Sweden; i Department of Paediatrics and Child Health, University of Cape Town, Cape Town, South Africa; j Department of Community Health and Rehabilitation, Umeå University, Sweden

**Keywords:** Congenital heart disease, Fatigue, MFI-20, Patient-reported outcome

## Abstract

**Background:**

Fatigue is a symptom that has been described among adult patients with congenital heart disease (CHD)**,** but the prevalence and impact of fatigue on the patient's daily life has been poorly studied. This study (i) examines the prevalence of fatigue in patients aged over 40 years with moderately complex or complex congenital heart disease compared to controls and (ii) explores the relationship between fatigue, heart disease complexity, clinical characteristics and self-reported New York Heart Association Functional Class (self-reported NYHA class).

**Methods:**

The Multidimensional Fatigue Inventory (MFI-20) was applied in 166 patients with moderately complex CHD or complex CHD (44 % females, median age 55.3 years, IQR 47.6–64.8) along with 89 controls (43 % female, median age 54.0, IQR 46.0–65.9). MFI-20 measured general fatigue, physical fatigue, mental fatigue, reduced motivation and reduced activity.

**Results:**

Physical fatigue (severe to very severe) was more common in complex CHD compared with moderately complex CHD (25 % *vs.* 52 %, *p* = 0.006). Complex CHD was associated with severe to very severe physical fatigue (odds ratio 3.1 (95 % CI 1.1–9.1). Patients with complex CHD had higher levels of self-reported NYHA class than patients with moderately complex CHD. All dimensions of fatigue were positively associated with self-reported NYHA class.

**Conclusions:**

Patients over the age of 40 with complex CHD were three times more likely to report severe to very severe physical fatigue and reported higher levels of self-reported NYHA class than patients with moderately complex CHD. This highlights the importance of considering CHD complexity in clinical practice.

## Highlights

• Fatigue has been described among adult patients with congenital heart disease (CHD).
• We examined fatigue in patients aged over 40 years with moderately complex and complex CHD.
• Physical fatigue (severe to very severe) was more common in complex CHD.
• Patients with complex CHD reported higher levels of self-reported NYHA class than those with moderately complex CHD.

## 1 Introduction

Almost 1 in 100 children are born with a congenital heart disease (CHD) [1] and in high-income countries 90–97 % of them are reported to reach adulthood [2,3]. Adults with CHD are a growing population as a result of advances in healthcare [4] and have outnumbered the children with CHD for many years [5]. Many of the adults with CHD are currently past middle-age [6] and in this growing and aging population, it is important to understand the patient's illness experiences and the long-term impact of living with CHD, to be able to provide appropriate healthcare [7].

The use of patient-reported outcomes (PROs) is increasing in the research of adults with CHD. Many perspectives of living with CHD, such as quality of life, physical functioning and mental health, have been covered in the Assessment of Patterns of Patient-Reported Outcomes in Adults with Congenital Heart Disease – International Study I (APPROACH-IS I) [[8], [9], [10]], but there are still knowledge gaps to fill. A PRO is defined as *“any report of the status of a patient's health condition, health behavior, or experience that comes directly from the patient, without interpretation of the patient's response by a clinician or anyone”* and can be used to understand the patient's subjective experience of a disease [11].

Fatigue is a symptom that is common among patients with heart disease [[12], [13], [14]] and can be described as an overwhelming and sustained feeling of exhaustion with a reduced capacity for mental and physical work [15]. Fatigue has been observed clinically in adults with CHD but the prevalence and impact of fatigue on the patient's daily life has been poorly studied in this group. Furthermore, fatigue is a complex, multidimensional, and subjective phenomenon [15] and has been associated with lower levels of health-related quality of life [12]. The Multidimensional Fatigue Inventory (MFI-20) is an instrument that allows patients to report fatigue in five dimensions: general fatigue, physical fatigue, mental fatigue, reduced motivation and reduced activity. Reduced motivation and reduced activity can be described as consequences of fatigue [16].

Based on basic anatomy and the chosen treatment method, CHD can be classified as simple, moderately complex or complex [17]. In a previous study [18], we have shown that in patients with complex and moderately complex CHD, the dimension “general fatigue” was the most prevalent and between 40 % to approximately 60 % of the patients reported severe to very severe general fatigue. The highest prevalence of severe to very severe general fatigue (60 %) was seen among patients with a complex CHD, (Fontan/total cavopulmonary connection [TCPC]). The study participants also reported a relatively high prevalence of physical fatigue, mental fatigue, and reduced activity. However, there was limited background data, no healthy controls included, and the study participants were relatively young (median age 36 years).

Therefore, the aim of the present study was to investigate the prevalence of fatigue in patients with moderately complex and complex CHD aged over 40 years and to compare them to healthy controls. A secondary objective was to explore associations between fatigue, heart defect complexity, clinical characteristics and self-reported NYHA class.

## 2 Methods

### 2.1 Study design and participants

The study used a cross-sectional design and was a Swedish sub-study of a global multicenter study, APPROACH-IS II [19]. Four centers in Sweden participated (Lund, Göteborg, Uppsala and Umeå). A total of 170 patients participated together with 93 controls who were recruited via the Total Population Register. However, four patients and four controls were excluded since they did not complete the MFI-20, leaving a total of 166 patients and 89 controls in this study.

Inclusion criteria were a confirmed CHD defined as “*a gross structural abnormality of the heart or intra-thoracic great vessels that is actually or potentially of functional significance”* [20] diagnosed before the age of 10 years, a complex or moderately complex CHD diagnosis, age over 40 years old, follow up at an adult CHD center or inclusion in a regional/national quality register and cognitive and language abilities to complete self-reported questionnaires. Patients who had undergone a heart transplant were excluded. The study was approved by the Ethics Review Authority in Sweden (Dnr 2019–06247). All participants gave their written informed consent**.** The study protocol conforms to the ethical guidelines of the 1975 Declaration of Helsinki.

### 2.2 Data collection

Data were collected from October 2020 to August 2022. All patients were recruited by telephone or when visiting the hospital for their routine clinic visit. Socio-demographic and medical variables were collected, and New York Heart Association functional class (NYHA class) and heart failure diagnosis or not were based on clinical assessment performed by a cardiologist. A range of PROs were collected including the MFI-20 and self-reported NYHA class.

### 2.3 The Multidimensional Fatigue Inventory (MFI-20)

Fatigue was assessed using the MFI-20 that enables the respondent to report the presence of fatigue according to five dimensions: general fatigue, physical fatigue, mental fatigue, reduced motivation and reduced activity. General fatigue includes general statements of fatigue, such as “I feel tired” or “I am rested”. Physical fatigue describes fatigue connected to physical functions, and mental fatigue refers to mental functions, such as difficulties in maintaining concentration. Reduced activity and reduced motivation can be described as consequences of fatigue [16]. Each dimension consists of four questions, which are answered on a five-degree Likert scale and which refer to the experience of fatigue in the last few days. The points from each question can be added up to a total score between 4 and 20 and can be categorized as none (4), mild (5–8), moderate (9-12) severe (13–16) and very severe (17–20) fatigue [13,14].

The reliability (internal consistency) of the MFI-20 was tested in our previous study and generated good-to-high values that ranged between 0.74 and 0.93 except for one low value in the dimension of “reduced motivation” (0.49) [18]. Other studies have also tested the reliability of the MFI-20 and reported good-to-high reliability for all subscales [16,21,22] except for some lower values in the dimension of “reduced motivation” [16,22]. The convergent validity of the MFI-20 has been tested and the correlation between the MFI-20 and Visual Analogue Scale (VAS) measuring fatigue ranged between 0.23 and 0.77 (p < 0.01) (16) and 0.32 to 0.62 (p < 0.001) [23].

### 2.4 Self-reported New York heart association functional class (self-reported NYHA class)

This measurement tool enables the patients to report their limitations in physical activity in four levels: 1) “I am not limited during physical activity”, 2) “I am slightly limited during physical activity”, 3) “I am considerably limited during physical activity” and 4) “I am very limited during physical activity” [19]. This tool was not applied in the control group.

### 2.5 Statistical analysis

Descriptive data are presented as frequencies with percentage and median with interquartile range (IQR). The results from the MFI-20 were dichotomized into none, mild and moderate vs. severe and very severe fatigue. Tests for differences between groups were performed with non-parametric tests: Mann-Whitney *U* test (two independent groups) or Kruskal-Wallis H-test (more than two independent groups). Ratios were tested with Chi^2^-test or Fisher's exact test when expected outcomes were low (<5) in any cell. Spearman's correlation analysis was used to test the relationship between two variables with skewed distribution and *r*_*s*_ of +1 indicating a perfect positive association, *r*_*s*_ of 0 indicating no association and *r*_*s*_ of −1 indicating a perfect negative association of ranks [24]. Logistic regression was used to test for associations in uni- and multivariable mode, the latter in a manual backward manner. Variables with p-values <0.05 were included in the multivariable model. The reliability (internal consistency) in terms of Cronbach's alpha was calculated for each group and dimension of fatigue. Values between 0.70 and 0.89 were considered to indicate good reliability and values above 0.90 high reliability [25]. Statistical analyzes were performed using the Statistical Package for Social Sciences version 28 (SPSS, IBM corp., Armonk, NY, USA). The null hypothesis was rejected for *p*-values <0.05.

## 3 Results

A total of 166 patients (median age 55.3 years, IQR 47.6–64.8) and 89 controls (median age 54.0 years, IQR 46.0–65.9) participated in the study. The patients with complex CHD (median age 48.1 years, IQR 43.1–54.9) were slightly younger than the patients with moderately complex CHD (median age 56.9 years, IQR 48.9–65.6), p < 0.001. There was no difference in sex distribution or body mass index (BMI) between the groups.

The hemoglobin saturation was lower in patients with complex CHD (96.5 %, IQR 93.0–98.0) compared to moderately complex CHD (98.0 %, IQR 97.0–99.0, *p* = 0.001) and compared to the controls (98.0 %, IQR 97.0–99.0 *p* < 0.001). There was no difference in the level of education or difference in their current work situation between the three groups (Table 1). There were higher numbers of patients with heart failure in the complex CHD group compared to the moderately complex group (40.9 % *vs*.14.9 %, *p* = 0.007). In the complex CHD group more patients were classified as NYHA classes II-III compared to the moderately complex group (65.2 % *vs*. 17.5 %, *p* < 0.001).

**Table 1:** Characteristics of the study participants.

	*p*-value∗	All CHD *n* = 166	Controls *n* = 89	Moderately complex CHD *n* = 143	Complex CHD *n* = 23	*p*-value∗
Age, *years*	0.86	55.3 (47.6–64.8)	54.0 (46.0–65.9)	56.9 (48.9–65.6)	48.1 (43.1–54.9)	0.003
Female (n, %)	0.84	73 (44.0)	38 (42.7)	61 (42.7)	12 (52.2)	0.68
BMI (kg/m^2^)	0.14	26.7 (24.0–29.6)	27.8 (24.5–30.7)	26.9 (24.1–29.4)	25.2 (23.8–31.9)	0.30
Haemoglobin saturation (%)∗	0.87	98.0 (97.0–99.0)^a^	98.0 (97.0–99.0)^b^	98.0 (97.0–99.0)^b^	96.5 (93.0–98.0)^c^	<0.001
**Highest achieved level of education∗ (n, %)**	0.10	e	d	e		0.25
Did not complete high school		19 (11.7)	6 (6.9)	17 (12.2)	2 (8.7)	
Graduated high school		83 (51.2)	40 (46.0)	70 (50.4)	13 (56.5)	
Bachelors degree or college degree		41 (25.3)	21 (24.1)	37 (26.6)	4 (17.4)	
Masters degree or higher		19 (11.7)	20 (23.0)	15 (10.8)	4 (17.4)	
**Current work situation? ∗ (n, %)**	0.45	e	c	f	c	0.083
Full-time paid job		84 (51.9)	54 (61.4)	70 (50.0)	14 (63.6)	
Part-time paid job		27 (16.7)	10 (11.2)	24 (17.1)	3 (13.6)	
Unemployed		2 (1.2)	0	2 (1.4)	0	
Job seeking		2 (1.2)	0	2 (1.4)	0	
Disability/government financial assistance		6 (3.7)	1 (1.1)	3 (2.1)	3 (13.6)	
Retired		38(23.5)	22 (25.0)	36 (25.7)	2 (9.1)	
Other		3(1.9)	1 (1.1)	3 (2.1)	0	

∗Age, BMI and saturation are presented as median and interquartile range (IQR).

∗Results of test of significance between all CHD and controls are presented in the left column. Mann-Whitney *U* test was used for test of significance in age, BMI and hemoglobin-saturation and Chi2 was used for test of significance in sex, highest achieved level of education and current work situation.

∗Results of test of significance between controls, moderately complex CHD and complex CHD are presented in the right column. Kruskal-Wallis H-test was used for test of significance in age, BMI and hemoglobin-saturation and Chi^2^ was used for test of significance in sex, highest achieved level of education and current work situation.

a Seven missing values.

b six missing values.

c one missing values.

d two missing values.

e four missing values.

f three missing values.

The diagnoses of the participants are presented in Supplementary Table 1. The most common diagnoses in the moderately complex CHD group were coarctation of the aorta, tetralogy of Fallot and aortic valve disease. Any variation of transposition of the great arteries was the most common diagnosis in the complex CHD group.

### 3.1 Fatigue

**Fig. 1:**
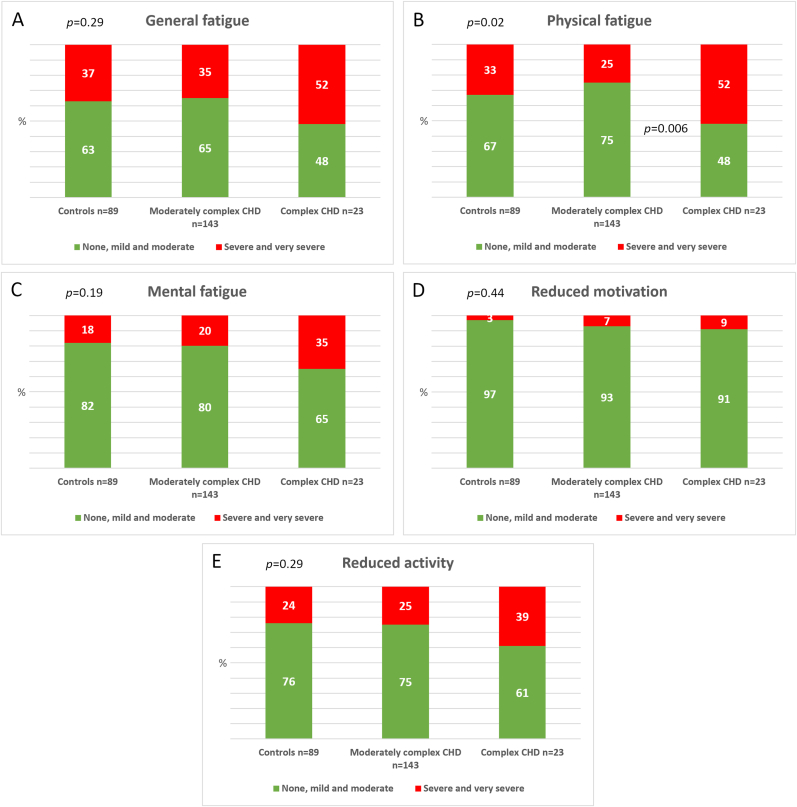
Fatigue scores dichotomized in none, mild, and moderate *vs.* severe and very severe in controls, moderately complex congenital heart disease (CHD) and complex CHD. A; General fatigue, B; Physical fatigue, C; mental fatigue, D; reduced motivation, E; reduced activity.

Fatigue scores were dichotomized in two categories: no, mild and moderate vs. severe and very severe fatigue. There were no differences in levels of severe to very severe fatigue in any of the dimensions between the controls and the CHD patients as one group. When comparing the control group, patients with moderately complex CHD and complex CHD (Fig. 1A–E), there was a difference in physical fatigue between patients with complex CHD and moderately complex CHD (52 % vs. 25 %, *p* = 0.006) (Fig. 1B).

In univariable logistic regression, patients with complex CHD (OR 3.4 95 % CI 1.4–9.3) and NYHA classes II-III (OR 2.7 95 % CI 1.2–5.8) were associated with severe to very severe physical fatigue. In a multivariable logistic regression, the OR for experiencing severe to very severe physical fatigue only persisted for complex CHD (OR 3.1 95 % CI 1.1–9.1) (Table 2).

**Table 2:** Univariable and multivariable logistic regression with severe to very severe physical fatigue as the dependent variable.

				Multivariate analysis					
	Univariate analysis			Initial model			Final model		
	**OR**	**95 % CI**	***p*-value**	**OR**	**95 % CI**	***p*-value**	**OR**	**95 % CI**	***p*-value**
**Complex CHD**	**3.4**	**1.4**–**8.3**	**0.008**	**3.1**	**1.1**–**9.1**	**0.04**	**3.4**	**1.4–8.3**	**0.008**
**Age**	1.01	0.98–1.04	0.60	1.02	0.98–1.06	0.31			
**Female**	1.5	0.8–2.9	0.25	1.7	0.8–3.5	0.20			
**Heart failure**	2.2	0.98–5.1	0.06	1.6	0.5–4.6	0.41			
**NYHA class > I**	**2.7**	**1.2**–**5.8**	**0.008**	1.4	0.5–3.8	0.50			

CI, confidence interval; CHD, congenital heart disease; OR, odds ratio; NYHAclass, New York Heart Association functional class. In the multivariate final model, manual backward analysis was performed.

### 3.2 Self-reported NYHA class

**Fig. 2:**
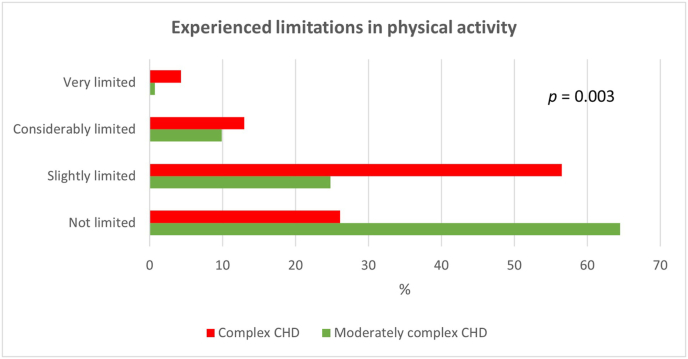
Self-reported New York Heart Association Functional Class; experienced limitations in physical activity. There was a difference between limitations in physical activity between patients with moderately complex congenital heart disease (CHD) and patients with complex CHD. There were two missing cases: the moderately complex CDH-group.

Patients with complex CHD experienced higher levels of limitations in physical activity compared to patients with moderately complex CHD (Fig. 2). All dimensions of fatigue were associated positively with experienced limitations in physical activity, general fatigue (*r*_s_ = 0.46, *p* < 0.001), physical fatigue (*r*_s_ = 0.48, *p* < 0.001), mental fatigue (*r*_s_ = 0.25, *p* = 0.001), reduced motivation (*r*_s_ = 0.32, *p* < 0.001) and reduced activity (*r*_s_ = 0.42, *p* < 0.001).

### 3.3 Reliability

The reliability (internal consistency) of the five subscales in the MFI-20 was tested with Cronbach's alpha for all three groups. Cronbach's alpha generated values of good or high reliability for all dimensions except in the dimension reduced motivation, 0.65 in the control group and 0.57 in the group with complex CHD, which are values of low reliability (Supplementary Table 2).

## 4 Discussion

This study showed that patients with complex CHD are about three times more likely to experience severe to very severe physical fatigue compared to those with moderately complex CHD, indicating that defect complexity is an important factor in experiencing physical fatigue. Furthermore, our findings indicate that patients with complex CHD experience higher levels of limitations in physical activity compared to patients with moderately complex CHD.

Previous research showed that among patients with heart failure physical fatigue is the most prevalent dimension [13,[26], [27], [28]] and higher levels of physical fatigue have also been associated with a higher NYHA class [13,27]. The group with complex CHD had a higher percentage of patients with heart failure and NYHA classes II-III than the group with moderately complex CHD. In our study, we did not find any associations between physical fatigue and heart failure. NYHA class was important in univariable analysis but disappeared in the multivariate mode.

When studying physical fatigue in the general Swedish population [29], there was no correlation between age and physical fatigue. Additionally, studies of fatigue in patients with heart failure and patients with pulmonary arterial hypertension did not find any associations between physical fatigue and age [13,14,27]. In this study we could not find any association between physical fatigue and age, but the patients with complex CHD were younger than the group with moderately complex CHD and had higher levels of physical fatigue.

Some studies suggest that women experience higher levels of physical fatigue than men [29] while others have reported no difference between men and women [14,30]. In our study, no association was found between the patient's sex and physical fatigue.

In our previous study with moderately complex and complex CDH [18], general fatigue was the most prevalent dimension compared to the other dimensions of fatigue, which was also the case in this study. The questions regarding general fatigue refers to the feeling of being tired, for example “I easily get tired” or “I feel tired”. The word “tired” may be easier to relate to than the word fatigue and this might explain the high prevalence of general fatigue. However, it is important to distinguish between the term “fatigue” and the term “tiredness”; fatigue does not disappear after a good night of sleep but tiredness does [15].

Patients with complex CHD reported higher levels of limitations in physical activity (self-reported NYHA class) compared to patients with moderately complex CHD. Physical fatigue was associated positively with experienced limitations in physical activity, indicating that when levels of physical fatigue increase, so do experienced limitations in physical activity. A qualitative study [31] described barriers for being physically active in adults with complex CHD as “being restricted by heart defect”, “feeling lethargic” and “having comorbidities”. These barriers were described in the category “energy levels”. Energy levels are likely to be affected by physical fatigue and experienced limitations in physical activity can most likely be seen as a consequence of physical fatigue in patients with complex CHD.

The levels of severe to very severe reduced motivation were low in all groups. This, along with our previous similar findings [18], suggests that reduced motivation might not be a major consequence of fatigue in adult patients with CHD.

The patients in the complex CHD group reported high levels of severe to very severe mental fatigue, which is consistent with our previous study [18]. The dimension of mental fatigue refers to difficulties in concentrating, and in clinical practice, many patients state that mental fatigue affects their ability to work and study. In this study, there was no difference in the level of education or current work situation between healthy controls, patients with moderately complex CHD and complex CHD. However, an international study [32] found that employment rates for adult patients with CHD are lower than in the general population in most countries and moderately complex and complex CHD were risk factors for work limitations among employed patients. Even if our study could not detect any differences in education or employment between the groups, they are important factors to consider in the context of mental fatigue and its consequences.

### 4.1 Study limitations

Firstly, patients with complex CHD were rather few but on the other hand reflective of the distribution of diagnoses in the adult CHD population. However, this might have affected the statistical strength in subgroup analysis. Secondly, this study was performed in a high-income health care system and is not necessarily applicable in other contexts. On the other hand, the study was a multicenter study which increases the generalizability of the results. Thirdly, participation in the study prolonged the patient's visit in the out-patient clinic by approximately 1–3 h and it is possible that the patients suffering from the highest levels of fatigue declined to participate. Fourthly, the reliability (Cronbach's alpha) was low in the dimension of reduced motivation in the control group and in the complex CHD group. Our previous study [18] as well as other studies [16,22,27] have also reported low values of reliability in this dimension which might indicate that the results from this dimension should be interpreted with caution. Fifthly, the cross-sectional design of this study precludes causal inference. Future studies using longitudinal or experimental designs are needed to better understand the causal pathways involved.

## 5 Conclusions

This study shows that patients over the age of 40 with complex CHD are approximately three times more likely to experience severe to very severe physical fatigue compared to those with moderately complex CHD. Additionally, our findings indicate that patients with complex CHD experience greater limitations in physical activity compared to those with moderately complex CHD.

These results underscore the importance of considering CHD complexity in clinical practice, as it is associated with both severe physical fatigue and activity limitations. This highlights the need for tailored support for patients with CHD. The MFI-20 may be a valuable tool for assessing fatigue in both complex and moderately complex CHD patients.

## CRediT authorship contribution statement

**Linda Ternrud:** Writing – review & editing, Writing – original draft, Visualization, Validation, Software, Resources, Project administration, Methodology, Investigation, Funding acquisition, Formal analysis, Data curation, Conceptualization. **Bengt Johansson:** Writing – review & editing, Visualization, Validation, Supervision, Software, Resources, Project administration, Methodology, Investigation, Funding acquisition, Formal analysis, Data curation, Conceptualization. **David Sparv:** Writing – review & editing, Visualization, Validation, Supervision, Software, Conceptualization. **Zacharias Mandalenakis:** Writing – review & editing, Visualization, Project administration, Investigation, Data curation. **Christina Christersson:** Writing – review & editing, Visualization, Project administration, Investigation, Data curation. **Liesbet Van Bulck:** Writing – review & editing, Software, Project administration, Methodology, Investigation, Data curation, Conceptualization. **Philip Moons:** Writing – review & editing, Software, Project administration, Methodology, Investigation, Data curation, Conceptualization. **Camilla Sandberg:** Writing – review & editing, Visualization, Validation, Supervision, Software, Project administration, Methodology, Investigation, Formal analysis, Data curation, Conceptualization. **Joanna Hlebowicz:** Writing – review & editing, Visualization, Validation, Supervision, Software, Resources, Project administration, Methodology, Investigation, Funding acquisition, Formal analysis, Data curation, Conceptualization.

## Declaration of competing interest

The authors declare the following financial interests/personal relationships which may be considered as potential competing interests: two of the authors (Zacharias Mandalenakis and Philip Moons) are IJC Congenital Heart Disease Editorial Board Members but had no involvement with the handling of this paper. If there are other authors, they declare that they have no known competing financial interests or personal relationships that could have appeared to influence the work reported in this paper.

## References

[1] Liu Y., Chen S., Zuhlke L., Black G.C., Choy M.K., Li N. (2019). Global birth prevalence of congenital heart defects 1970-2017: updated systematic review and meta-analysis of 260 studies. Int J Epidemiol.

[2] Moons P., Bovijn L., Budts W., Belmans A., Gewillig M. (2010). Temporal trends in survival to adulthood among patients born with congenital heart disease from 1970 to 1992 in Belgium. Circulation.

[3] Mandalenakis Z., Rosengren A., Skoglund K., Lappas G., Eriksson P., Dellborg M. (2017). Survivorship in children and young adults with congenital heart disease in Sweden. JAMA Intern Med.

[4] van der Linde D., Konings E.E., Slager M.A., Witsenburg M., Helbing W.A., Takkenberg J.J. (2011). Birth prevalence of congenital heart disease worldwide: a systematic review and meta-analysis. J Am Coll Cardiol.

[5] Marelli A.J., Ionescu-Ittu R., Mackie A.S., Guo L., Dendukuri N., Kaouache M. (2014). Lifetime prevalence of congenital heart disease in the general population from 2000 to 2010. Circulation.

[6] Bhatt A.B., Foster E., Kuehl K., Alpert J., Brabeck S., Crumb S. (2015). Congenital heart disease in the older adult: a scientific statement from the American Heart Association. Circulation.

[7] Goossens E., Fleck D., Canobbio M.M., Harrison J.L., Moons P. (2013). Development of an international research agenda for adult congenital heart disease nursing. Eur J Cardiovasc Nurs.

[8] Apers S., Kovacs A.H., Luyckx K., Alday L., Berghammer M., Budts W. (2015). Assessment of Patterns of patient-reported outcomes in adults with congenital heart disease - international study (APPROACH-IS): rationale, design, and methods. Int J Cardiol.

[9] Apers S., Kovacs A.H., Luyckx K., Thomet C., Budts W., Enomoto J. (2016). Quality of life of adults with congenital heart disease in 15 countries: evaluating country-specific characteristics. J Am Coll Cardiol.

[10] Moons P., Luyckx K., Thomet C., Budts W., Enomoto J., Sluman M.A. (2021). Physical functioning, mental health, and quality of life in different congenital heart defects: comparative analysis in 3538 patients from 15 countries. Can J Cardiol.

[11] US Department of Health and Human Service Food and Drug Administration. (2009). *Guidance for industry. Patient-reported outcome measures: use in medical product development to support labeling claims*. Silver Spring MD: Food and Drug Administration.

[12] Staniute M., Bunevicius A., Brozaitiene J., Bunevicius R. (2014). Relationship of health-related quality of life with fatigue and exercise capacity in patients with coronary artery disease. Eur J Cardiovasc Nurs.

[13] Falk K., Swedberg K., Gaston-Johansson F., I (2007). Ekman Fatigue is a prevalent and severe symptom associated with uncertainty and sense of coherence in patients with chronic heart failure. Eur J Cardiovasc Nurs.

[14] Tartavoulle T.M., Karpinski A.C., Aubin A., Kluger B.M., Distler O., Saketkoo L.A. (2018). Multidimensional fatigue in pulmonary hypertension: prevalence, severity and predictors. ERJ Open Res.

[15] Tiesinga L.J., Dassen T.W., Halfens R.J. (1996). Fatigue: a summary of the definitions, dimensions, and indicators. Nurs Diagn.

[16] Smets E.M.A., Garssen B., Cull A., de Haes J.C. (1996). Application of the multidimensional fatigue inventory (MFI-20) in cancer patients receiving radiotherapy. Br J Cancer.

[17] Stout K.K., Daniels C.J., Aboulhosn J.A., Bozkurt B., Broberg C.S., Colman J.M. (2019). 2018 AHA/ACC guideline for the management of adults with congenital heart disease: a report of the American college of cardiology/American heart association task force on clinical practice guidelines. J Am Coll Cardiol.

[18] Ternrud L., Hlebowicz J., Sandberg C., Johansson B., Sparv D. (2021). Prevalence of fatigue in adults with congenital heart disease. Cardiol Young. Oct.

[19] Van Bulck L., Kovacs A.H., Goossens E., Luyckx K., Zaidi A., Wang J.K. (2022). Rationale, design and methodology of APPROACH-IS II: international study of patient-reported outcomes and frailty phenotyping in adults with congenital heart disease. Int J Cardiol.

[20] Mitchell S.C., Korones S.B., Berendes H.W. (1971). Congenital heart disease in 56109 births. Circulation.

[21] Furst C.J., Ahsberg E. (2001). Dimensions of fatigue during radiotherapy. An application of the multidimensional fatigue inventory. Support Care Cancer.

[22] Hagelin C.L., Wengström Y., Runesdotter S., Furst C.J. (2007). The psychometric properties of the Swedish Multidimensional Fatigue Inventory MFI-20 in four different populations. Acta Oncol.

[23] Ericsson A., Mannerkorpi K. (2007). Assessment of fatigue in patients with fibromyalgia and chronic widespread pain. Reliability and validity of the Swedish version of the MFI-20. Disabil Rehabil.

[24] Björk J. (2020).

[25] Olsson H.S.S. (2011). Forskningsprocessen.

[26] Falk K., Patel H., Swedberg K., Ekman I. (2009). Fatigue in patients with chronic heart failure - a burden associated with emotional and symptom distress. Eur J Cardiovasc Nurs.

[27] Higa H., Lennie T.A., Chung M.L., Tsuchihashi-Makaya M. (2023). Associations of multidimensional fatigue with the physical, psychological, and situational factors in outpatients with heart failure: a cross-sectional study. Eur J Cardiovasc Nurs.

[28] Hägglund L., Boman K., Olofsson M., Brulin C. (2007). Fatigue and health-related quality of life in elderly patients with and without heart failure in primary healthcare. Eur J Cardiovasc Nurs.

[29] Engberg I., Segerstedt J., Waller G., Wennberg P., Eliasson M. (2017). Fatigue in the general population- associations to age, sex, socioeconomic status, physical activity, sitting time and self-rated health: the northern Sweden MONICA study 2014. BMC Public Health.

[30] Falk K., Swedberg K., Gaston-Johansson F., Ekman I. (2006). Fatigue and anaemia in patients with chronic heart failure. Eur J Heart Fail.

[31] Bay A., Lämås K., Berghammer M., Sandberg C., Johansson B. (2021). Enablers and barriers for being physically active: experiences from adults with congenital heart disease. Eur J Cardiovasc Nurs.

[32] Sluman M.A., Apers S., Sluiter J.K., Nieuwenhuijsen K., Moons P., Luyckx K. (2019). Education as important predictor for successful employment in adults with congenital heart disease worldwide. Congenit Heart Dis.

