# Supplementary material for: Fatigue in adults with congenital heart disease aged over 40 years
Source: Int J Cardiol Congenit Heart Dis. 2025 Jun 16;21:100601. doi: 10.1016/j.ijcchd.2025.100601 (PMC12280406; doi:10.1016/j.ijcchd.2025.100601)
Supplement: Multimedia component 1 [file mmc1.docx]

| **Diagnosis** | ***n*** | **%** |
| --- | --- | --- |
| **Moderately complex CHD** | **143** |  |
| AVSD (Partial or complete) | 9 | 5.4 |
| Congenital aortic valve disease | 26 | 15.7 |
| Coarctation of the aorta | 55 | 33.1 |
| Ebstein’s anomaly | 6 | 3.6 |
| Infundibular right ventricular outflow obstruction | 1 | 0.6 |
| Ostium primum ASD | 1 | 0.6 |
| Moderate or large persistent ductus arteriosus | 1 | 0.6 |
| Pulmonary valve regurgitation (moderate or greater) | 2 | 1.2 |
| Pulmonary valve stenosis (moderate or greater) | 7 | 4.2 |
| Sinus venosus defect | 2 | 1.2 |
| Subvalvular aortic stenosis | 2 | 1.2 |
| Supravalvular aortic stenosis | 1 | 0.6 |
| Repaired tetralogy of Fallot | 27 | 16.3 |
| VSD with associated abnormality and/or moderate or greater shunt | 2 | 1.2 |
| Other defect of moderate complexity | 1 | 0.6 |
| **Complex CHD** | **23** |  |
| Double-outlet ventricle | 1 | 0.6 |
| Fontan circulation | 3 | 1.8 |
| Single ventricle | 1 | 0.6 |
| Pulmonary atresia | 3 | 1.8 |
| TGA (All forms) | 15 | 9.0 |

**Supplementary table 1. Diagnoses of the study participants.**

ASD, atrial septal defect; AVSD, atrioventricular septal defect; CHD, congenital heart disease; TGA, transposition of the great arteries; VSD, ventricular septal defect
