# Supplementary material for: Fatigue in adults with congenital heart disease aged over 40 years
Source: Int J Cardiol Congenit Heart Dis. 2025 Jun 16;21:100601. doi: 10.1016/j.ijcchd.2025.100601 (PMC12280406; doi:10.1016/j.ijcchd.2025.100601)
Supplement: Multimedia component 2 [file mmc2.docx]

**Supplementary table 2. Reliability of MFI-20**

|  | **Controls**  **n = 89** | **Moderately complex CHD**  **n = 146** | **Complex CHD**  **n = 23** |
| --- | --- | --- | --- |
| General fatigue | 0.84 | 0.78 | 0.85 |
| Physical fatigue | 0.83 | 0.88 | 0.87 |
| Mental fatigue | 0.77 | 0.78 | 0.79 |
| Reduced motivation | 0.65 | 0.72 | 0.57 |
| Reduced activity | 0.86 | 0.86 | 0.92 |

Reliability (internal consistency) is measured with Cronbach’s Alpha. Values between

0.70 and 0.89 indicate good reliability and values above 0.90 high reliability (25).
